# Validity and reliability of the Turkish version of the Eating Disorder Examination Questionnaire (EDE-Q-13): short-form of EDE-Q

**DOI:** 10.1186/s40337-022-00628-4

**Published:** 2022-07-14

**Authors:** Kübra Esin, Feride Ayyıldız

**Affiliations:** 1 Department of Nutrition and Dietetics, Faculty of Health Sciences, Gaziosmanpaşa University, Tokat, Turkey; 2 Department of Nutrition and Dietetics, Faculty of Health Sciences, Gazi University, Ankara, Turkey

**Keywords:** EDE-Q, Eating disorder, EDE-Q-13, Body appreciation, Life satisfaction

## Abstract

**Background:**

The Eating Disorder Examination Questionnaire (EDE-Q) is a frequently used scale to evaluate eating behaviors and attitudes. In recent years, its use has increased due to the fact that the use of short forms is more practical. The aim of this study was to validate the short Turkish form of the Eating Disorder Examination Questionnaire (EDE-Q) including 13 items.

**Methods:**

The study included 924 adults at a mean age of 30.3 ± 10.93 years. EDEQ-13 was translated and adapted to Turkish according to the Beaton Guidelines. The Eating Attitudes Test-26 (EAT-26), the Satisfaction with Life Scale (SWLS), and the Body Appreciation Scale (BAS) were used to analyze their relationships to EDE-Q-13.

**Results:**

In this study, the rate of the total variance explained by the factors of EDE-Q-13 according to the Explanatory Factor Analysis (EFA) results of the scale was 83.54%. The Cronbach's alpha value of the scale was 0.89, and the Cronbach's alpha values of the 5 subscales were calculated in the range of 0.75–0.94. The criterion validity analysis showed an acceptable correlation between EDE-Q-13 and EAT-26, SWLS, and BAS. The confirmatory factor analysis (CFA) revealed that the model had fit values that were acceptable or good.

**Conclusion:**

Both EFA and CFA results showed that it is appropriate to use the Turkish EDE-Q-13. EDE-Q-13 was significantly correlated with eating disturbances, body appreciation, and life satisfaction. In conclusion, the Turkish version of EDE-Q-13 possesses high levels of validity and reliability.

## Plain English summary

The rate of eating disorders has been increasing in recent years. Although there are many scales in the evaluation of eating disorders, one of the most frequently used scales is the Eating Disorder Examination Questionnaire (EDE-Q). In recent years, its use has increased due to the fact that the use of short forms is more practical. The aim of this study was to validate the short Turkish form of EDE-Q. The study included 924 adults. Our results showed that it is appropriate to use the Turkish EDE-Q-13. EDE-Q-13 was significantly correlated with eating disturbances, body appreciation, and life satisfaction. In conclusion, the Turkish version of EDE-Q-13 possesses high levels of validity and reliability.

## Background

Eating disorders are a group of chronic psychiatric diseases characterized by severe disturbances in eating behaviors originating from the interactions of genetic, biological, psychological, and socio-cultural factors [1]. Although eating disorders are more common in Western societies, the incidence of these disorders has increased recently in both developed and developing countries [2, 3]. The increase in the incidence and prevalence of eating disorders, their comorbidities, tendency to become chronic, and high risk of mortality requires a detailed and accurate evaluation of these conditions [4].

Various scales are used in the evaluation of the variety and severity of eating disorder symptoms, and the most widely used instrument among these scales is the Eating Disorder Examination Questionnaire (EDE-Q), which is a 28-item self-report instrument developed by Fairburn and Beglin [5]. EDE-Q has been used to evaluate the attitudinal and behavioral symptoms of eating disorders for more than a quarter century [5], and its validity and reliability have been established in many languages [6–12], including Turkish [13]. EDE-Q consists of four subscales: Restraint, Eating Concern, Weight Concern, and Shape Concern [5]. However, studies examining the factor structure of the scale in different populations have not supported the 4-factor construct [14–16]. Some confirmatory factor analyses have shown that Shape and Weight Concern might be represented better as a single factor and suggested a 3-factor construct instead of the original 4-factor construct [17–19].

Shorter versions of EDE-Q have been developed because the 4-factor structure of the original questionnaire was not confirmed. It was also not economical in terms of time due to the large number of items [20, 21]. Therefore, 18-item [17], 12-item [22], 11-item [23], 8-item [24], and 7-item [18] versions of the scale have been developed. Researchers who wanted to use a short version of the scale have explored the most productive version. Among the short versions of EDE-Q, the 3-factor EDE-Q-7 (Dietary Restraint, Shape and Weight Over-Evaluation, Body Dissatisfaction) yielded excellent psychometric properties; however, it might yield incomplete data because it does not include items about "binge eating" and "purging", which are important in evaluating the symptoms of eating disorders [20]. Therefore, the 13-item 5-factor short-form of EDE-Q-13 was developed by Lev-Ari et al. [25]. In addition to the 7 items of the original EDE-Q-7, this scale includes 6 more items, all of which are related to binge eating and purging. The subscales of EDE-Q-13 consist of Eating Restraint, Shape and Weight Over-Evaluation, Body Dissatisfaction, Bingeing, and Purging [25]. Thus, EDE-Q-13 contains both the 3-factor construct of EDE-Q with high psychometric qualities and the Bingeing and Purging factors that are missing in the other short versions. EDE-Q-13 was reported to be a concise, user-friendly, and reliable measurement tool for identifying eating disorder symptoms in clinical and community samples [25]. A strong positive correlation was observed between EDE-Q-13 and the original EDE-Q. Additionally, EDE-Q-13 showed a high correlation with other eating disorder measures and measures of accompanying psychopathologies [25].

Considering that there is an alarming increase in eating disorders in Turkey, especially in young people [26], it is important and necessary to ensure the cultural validity and reliability of eating disorder measurement instruments. Although the long form of the frequently used EDE-Q has validity and reliability in Turkish, there exists no study on the short form of the Turkish version. EDE-Q-13, a short, user-friendly, and reliable measurement instrument, will make it easier to measure the symptoms of eating disorders in clinical, epidemiological, and fundamental studies. This study aimed to determine the validity and reliability of EDE-Q-13 in Turkish.

## Methods

### Participants

All participants were Turkish-speaking and Turkish national adults living in Turkey. Non-Turkish individuals, non-adult individuals, and those who did not agree to participate were not included in the study. Simple random sampling was used to select participants. Before the study, a power analysis was performed using the G*Power software to determine the number of individuals to be included in the sample. Based on alpha (α) = 0.05, power (1 − β) = 0.80, and the considered effect size, the minimum required sample size was calculated as 560 participants, and it was decided that at least 616 individuals should be recruited with the possibility of 10% data loss. A questionnaire was used as the data collection tool. The questionnaire was disseminated via e-mail or WhatsApp to the individuals who agreed to participate in the study. The participants were able to fill out the online questionnaire after they agreed to participate in the study.

The study included 924 participants (35.3% male and 64.7% female) between the ages of 18 and 64. Their mean age was 30.3 ± 10.93 years, 42.0% of the participants were students, and 20.3% worked in the private sector. The mean BMI of the participants was 24.3 ± 4.51 kg/m^2^ (Male: 25.8 ± 3.69, Female: 23.5 ± 4.71), and 52.5% had a normal body weight according to the BMI classification. The test–retest reliability coefficient of the scale was found to be 0.93.

### Measurements

#### EDE-Q-13

EDE-Q was developed by Fairburn and Beglin [5] to evaluate eating disorders and consists of 28 items. It evaluates eating behaviors during 28 days in 4 subscales: Restraint, Shape Concern, Weight Concern, and Eating Concern. The validity and reliability study of the Turkish version of the scale in adolescents was performed by Yucel et al. [13]. Each item in EDE-Q is scored on a 7-point Likert-type scale (0 = never, 1 = 1–5 days, 2 = 6–12 days, 3 = 13–15 days, 4 = 16–22 days, 5 = 23–27 days, 6 = every day). The scores of the scale are evaluated based on the sum of the scores of the items in the entire scale or each subscale of the scale, and higher scores indicate higher levels of eating-related psychopathology [5].

EDE-Q-13 consists of 13 items and 5 subscales, namely Eating Restraint (ER), Shape and Weight Over-Evaluation (SWO), Body Dissatisfaction (BD), Bingeing, and Purging [25]. Its validity study was conducted by Lev-Ari et al. [25], and the scale showed a strong positive correlation with the original EDE-Q. The Cronbach's alpha values for the subscales of EDE-Q-13 were reported as 0.99 for SWO, 0.89 for BD, 0.92 for ER, 0.89 for Bingeing, and 0.63 for Purging.

#### EAT-26

The Eating Attitudes Test—Short Form (EAT-26) was developed by Garner et al. [27]. Ergüney-Okumuş et al. [28] conducted the validity and reliability study of the Turkish version of EAT-26 and reported a Cronbach's alpha value of 0.84. The evaluation of EAT-26 scores is made based on the total score of items scored according to a 6-point Likert-type scale, and item 26 is scored in reverse. A higher total EAT-26 score indicates a higher risk of eating disorders. In this study, the Cronbach's alpha coefficient of EAT-26 was measured as 0.91.

#### SWLS

In 1985, Diener et al. [29] developed a valid and reliable measurement instrument to examine satisfaction with life. The Satisfaction with Life Scale (SWLS) is a one-dimensional and 5-item measurement instrument. Increasing scale scores indicate increasing satisfaction with life. Its validity and reliability study in Turkish was performed by Dağlı et al. [30]. Each item is scored on a 5-point Likert-type scale with response options from 1 = Strongly disagree to 5 = Strongly agree. The Cronbach’s alpha internal consistency coefficient of the Turkish version was reported as 0.88, and the test–retest reliability coefficient was 0.97. In this study, the Cronbach's alpha coefficient of the scale was found to be 0.88.

#### BAS

The Body Appreciation Scale (BAS) was developed by Avalos et al. [31] to measure the extent to which an individual is satisfied with their body, accepts their body as-is, and takes care of it. Scoring is made based on a 5-point Likert-type scale (1 = Never, 5 = Always). Higher total scores on this 13-item scale indicate greater body appreciation.

The validity and reliability study of the scale in Turkish was performed by Bakalım et al. [32]. The Turkish version of the scale consists of 9 items and two subscales. The results of a competing model analysis showed that a two-factor model (Factor 1 = General Body Appreciation; Factor 2 = Body Image Investment) with four items deleted was the best of the proposed models. The Cronbach's alpha coefficient of the scale was reported as 0.94, and the test–retest reliability coefficient was 0.90 in the Turkish version. In this study, the Cronbach's alpha coefficient was found 0.94.

### Body mass index (BMI)

The height and body weight values of the participants were recorded based on self-report. Body Mass Index (BMI) values were calculated with the following equation: 'BMI = Body weight (kg)/(height (m))^2^. According to the World Health Organization (WHO) [33], BMI is classified as: < 18.50 kg/m^2^ (underweight), 18.50–24.99 kg/m^2^ (normal weight), 25.00–29.99 kg/m^2^ (pre-obese), and ≥ 30.00 kg/m^2^ (obese).

### Procedure

The demographic characteristics of the participants, their age, height, and body weight were recorded based on self-report. Several scales with established validity and reliability in Turkish were used to determine the criterion validity of the scale, including the Eating Attitudes Test-Short Form (EAT-26) [27, 28], the Satisfaction with Life Scale (SWLS) [29, 30], and the Body Appreciation Scale (BAS) [31, 32].

For the validity and reliability study of the Turkish EDE-Q-13, permission was obtained from Lev-Ari [25] via e-mail. The questionnaire was then translated according to the guidelines created by Beaton et al. [34]. The original English version of the questionnaire was translated into Turkish by two independent translators, who speak both Turkish and English. One of the translators has a medical/clinical background, and the other has no such background. A single Turkish questionnaire was created by evaluating the two translations. This Turkish version was translated back into English by two native English speakers with a good command of Turkish, and their back-translations were compared to the original translation version. The Turkish form of the scale was finalized by a team of translators and researchers. For the retest, thirty respondents were selected randomly, and they were asked to fill out the questionnaire again after 15 days.

### Statistical analysis

The data obtained in this study were analyzed using the SPSS (Statistical Package for the Social Sciences) for Windows 25.0 program. Descriptive statistical methods were used for data analysis. To determine the normality of the distribution of the data, Skewness and Kurtosis Tests and Z-values were used. The Cronbach’s alpha value was calculated using the SPSS program to determine internal consistency indicating reliability. A value of Cronbach's alpha should be at least 0.60 to be acceptable, and a good value is considered to be 0.70 or above [35]. A *p*-value smaller than 0.05 was considered statistically significant.

Explanatory Factor Analysis (EFA) and Confirmatory Factor Analysis (CFA) were performed to assess the reliability and construct validity of the adapted scale. The CFA in this study was performed using the AMOS-24 software. The following parameters were examined within the scope of CFA: Kaiser–Meyer–Olkin (KMO) value and multiple fit indices including Root Mean Square Error of Approximation (RMSEA), Goodness-of-Fit Index (GFI), Adjusted Goodness-of-Fit Index (AGFI), Comparative Fit Index (CFI), Normed Fit Index (NFI), Tucker–Lewis Index (TLI). In CFA, χ^2^/df ≤ 3.0, RMSEA ≤ 0.05, 0.90 ≤ GFI, 0.95 ≤ AGFI, 0.95 ≤ CFI, 0.95 ≤ NFI and 0.95 ≤ TLI indicate good fit, and 3 ≤ χ^2^/df ≤ 5, 0.05 ≤ RMSEA ≤ 0.08, 0.80 ≤ GFI ≤ 0.90, 0.85 ≤ AGFI ≤ 0.95, 0.85 ≤ CFI ≤ 0.95, 0.80 ≤ NFI ≤ 0.95, and 0.80 ≤ TLI ≤ 0.95 indicate acceptable fit according to conventions for Model Confirmatory Factor Analysis Fit Indices [36].

## Results

### Explanatory factor analysis and reliability

The results of the EFA and Cronbach’s alpha internal consistency coefficient of EDE-Q-13 are presented in Table 1. Before the EFA, the KMO test was performed to test the suitability of the sample size for factor analysis. As a result of the analysis, the KMO value was determined as 0.84. In line with this finding, it was concluded that the sample size was adequate for factor analysis [37]. In the EFA, the acceptable level for factor loading values was determined as 0.40 [35, 38]. Additionally, in the results of the Bartlett’s test of sphericity, the Chi-squared value was found significant (χ^2^(78) = 8694.05; KMO = 0.84 *p* < 0.01). Accordingly, it was assumed that the data had multivariate normal distribution. The total variance explained by the factors was 83.54%.

**Table 1:** The results of the EFA and Cronbach’s alpha internal consistency analysis of EDE-Q-13

Factors and items	Total variance (%)	Factor loading	X ± SD
F1 (α = 0.92)	21.78		1.7 ± 1.85
I1		0.88	1.97 ± 2.01
I2		0.86	1.6 ± 1.96
I3		0.89	1.7 ± 1.98
F2 (α = 0.94)	12.01		2.1 ± 2.05
I4		0.77	2.0 ± 2.11
I5		0.77	2.1 ± 2.11
F3 (α = 0.93)	15.29		2.5 ± 2.22
I6		0.89	2.5 ± 2.30
I7		0.86	2.5 ± 2.29
F4 (α = 0.84)	18.13		1.1 ± 1.30
I8		0.78	1.0 ± 1.48
I9		0.84	1.2 ± 1.56
I10		0.81	1.0 ± 1.43
F5 (α = 0.75)	16.32		0.3 ± 0.84
I11		0.83	0.2 ± 0.94
I12		0.87	0.2 ± 0.84
I13		0.70	0.5 ± 1.24
Toplam score (α = 0.89)			3.8 ± 3.09

Kaiser–Meyer–Olkin (KMO) = 0.84; χ^2^ (78) = 8694.05; Barlett test of sphericity (*p*) < 0.01

F: Factor, I: Item; F1: Eating Restraint, F2: Shape and Weight Over-evaluation, F3: Body Dissatisfaction, F4: Bingeing, F5: Purging

A Cronbach’s alpha coefficient above 0.70 is considered sufficient for reliability. In this study, as seen in Table 2 the Cronbach's alpha coefficient of the scale was determined as 0.89, and the Cronbach's alpha values of the 5 subscales were calculated in the range of 0.75–0.94. In this case, it was determined that the reliability of the adapted scale, and all its subscales, was high. The test–retest reliability coefficient of the scale was found to be 0.93.

**Table 2:** Correlations between the EDE-Q-13 subscales and their correlations with EAT-26, BAS, SWLS, and BMI

	Mean	SD	1	2	3	4	5	6	7	8	9	10	11	12
1. Total EDE-Q-13	3.8	3.09	1											
2. ER	1.7	1.85	0.80*	1										
3. SWO	2.1	2.05	0.84*	0.63*	1									
4. BD	2.5	2.22	0.78*	0.45*	0.66*	1								
5. Bingeing	1.1	1.30	0.71*	0.42*	0.47*	0.46*	1							
6. Purging	0.3	0.84	0.51*	0.36*	0.34*	0.26*	0.41*	1						
7. EAT-26	12.7	12.81	0.39*	0.34*	0.37*	0.27*	0.23*	0.25*	1					
8. Total BAS	31.3	8.56	− 0.42*	− 0.19*	− 0.39*	− 0.47*	− 0.36*	− 0.24*	− 0.28*	1				
9. General Body Appreciation	24.6	6.78	− 0.40*	− 0.16*	− 0.36*	− 0.46*	− 0.34*	− 0.21*	− 0.24*	0.98*	1			
10. Body Image Investment	6.6	2.25	− 0.41*	− 0.25*	− 0.38*	− 0.37*	− 0.33*	− 0.27*	− 0.33*	0.83*	0.72*	1		
11. SWLS	15.0	4.29	− 0.07**	0.05	− 0.09*	− 0.12*	− 0.12*	− 0.08*	− 0.08*	0.45*	0.47*	0.33*	1	
12. BMI	24.3	4.51	0.25*	0.16*	0.15*	0.29*	0.22*	0.10*	0.07*	− 0.27*	− 0.28*	− 0.19*	0.10	1

r: Pearson/Spearman correlation coefficient *p < 0.01 and **p < 0.05

EDE-Q, Eating Disorder Examination Questionnaire; ER, Eating Restraint; SWO, Shape and Weight Over-Evaluation; BD, Body Dissatisfaction; EAT-26, The Eating Attitudes Test-26; BAS, Body Appreciation Scale; SWLS, Satisfaction with Life Scale; BMI, Body Mass Index

### Confirmatory factor analysis (CFA)

The model goodness-of-fit statistics of the scale were found as follows: GFI = 0.95, CFI = 0.97, NFI = 0.96, and TLI = 0.96. These statistics were found to show good fit based on recommended thresholds. The other indices reached acceptable fit levels as: RMSEA = 0.06, χ^2^/df = 4.90, and AGFI = 0.93.

**Fig. 1:**
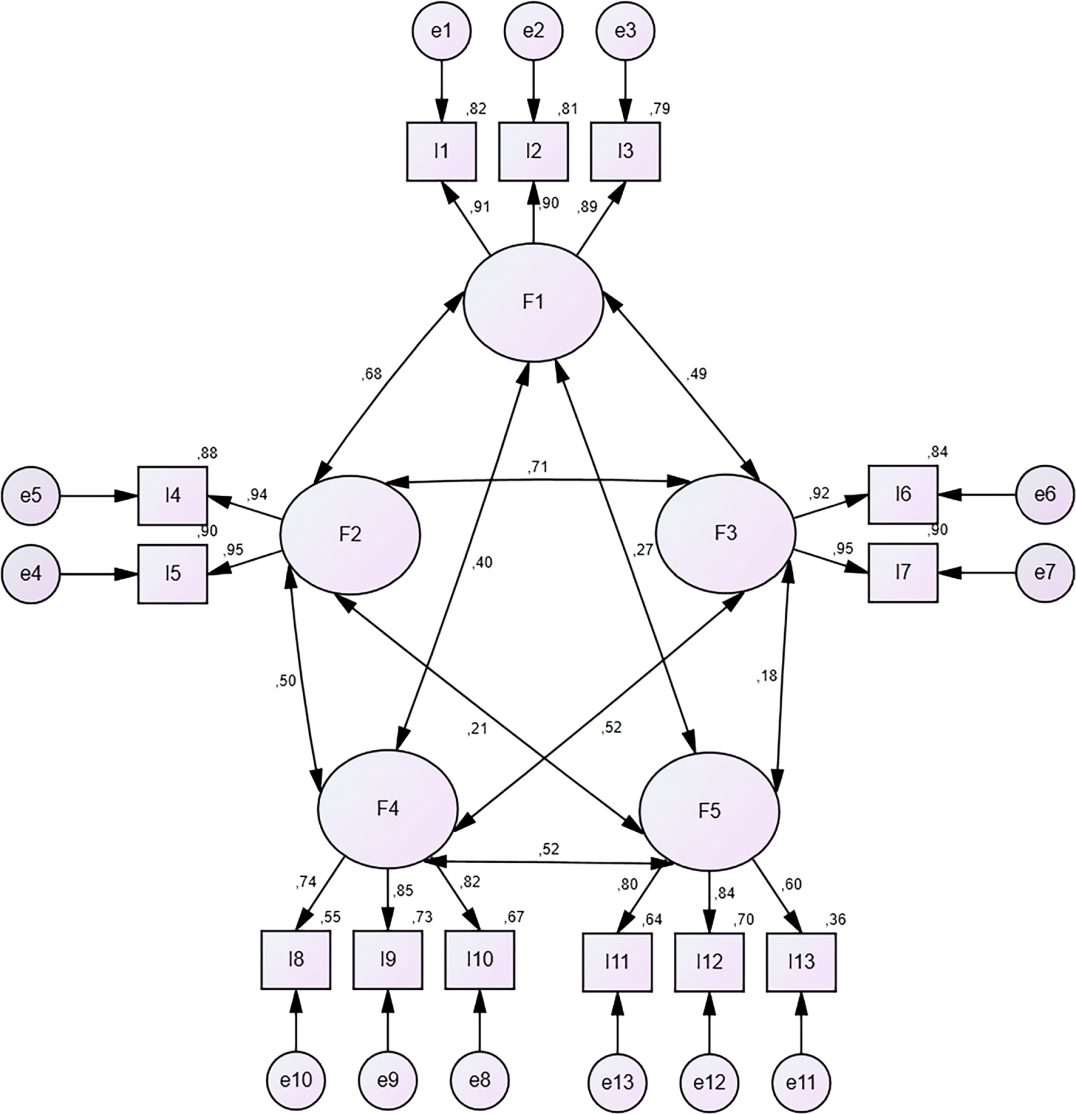
Confirmatory factor analysis of EDE-Q-13. F1: eating restraint, F2: shape and weight over-evaluation, F3: body dissatisfaction, F4: bingeing, F5: purging

The t-statistics for the items showed that all items were significant. The accepted limit for factor loading values was determined as 0.40. In the analyses, no item with a score below 0.40 was identified, and the factor loadings were within acceptable limits. The CFA results of EDE-Q-13 are shown in Fig. 1.

### Criterion validity

Correlations between the EDE-Q-13 subscales and EAT-26, BAS, SWLS, and BMI are given in Table 2. There was a significant positive correlation between the EDE-Q-13 scores of the participants and their EAT-26 (including its subscales) scores and BMI values. There was a significant negative correlation between the EDE-Q-13 scores of the participants and their BAS (including its subscales) scores and SWLS scores. The BMI values, EDE-Q-13 total scores, and EDE-Q-13 subscale scores of the participants were correlated positively with their EAT-26 scores and negatively with their BAS and SWLS scores.

## Discussion

This study was conducted to establish the validity and reliability of the Turkish version of EDE-Q-13, which was originally developed by Lev-Ari [25]. The relationship of EDE-Q-13 with scales evaluating eating behaviors, life satisfaction, and body appreciation was analyzed. The structure of the scale was evaluated using EFA and CFA, and the correlations between the related variables were investigated. According to the results, the Turkish version of EDE-Q-13 provided high levels of validity and reliability. And also The Turkish version of EDE-Q-13 was significantly correlated with eating disturbances, body appreciation, and life satisfaction.

The results of the present study support the original 13-item and 5-factor structure [25] of EDE-Q-13, including Eating Restraint, Shape and Weight Over-Evaluation, Body Dissatisfaction, Bingeing, and Purging, as opposed to the initially developed EDE-Q that consisted of 4 factors. Some studies have also not supported the 4-factor model of EDE-Q [14–16]. Among these studies, some have suggested a 3-factor construct instead of the original 4-factor model [17–19]. Contrary to other short versions of EDE-Q [17, 18, 22–24], the positive side of EDE-Q-13 has been reported as that it contains items about binge eating and purging [20, 25]. The major disadvantage of EDE-Q to date was that Bingeing and Purging items were in the form of open-ended questions and not included in the scoring process. Having Bingeing and Purging subscales in EDE-Q-13 and including them in scoring can be considered superior properties of EDE-Q-13 over other short versions [25]. This is also a positive characteristic for its use in clinical and community samples [25].

CFA values and construct validity of short versions of EDE-Q are very important. In this study, according to recommended thresholds in the literature, the goodness-of-fit index values of the scale were determined to indicate a good or acceptable fit. In contrast, for EDE-Q-8 [24] and EDE-Q-18 [17], while certain fit indices were within recommended thresholds, the RMSEA values were deemed unacceptable, thus not supporting their proposed constructs. Some scales such as the modified EDE-Q-7 “reflect less overlap and redundancy” than the original EDE-Q [19]. It is important that CFA values of EDE-Q versions and their compatibility with other indicators (e.g., eating attitudes, life satisfaction) are provided, as well as their practicality and brevity like EDE-Q-13.

This study examined the properties of EDE-Q-13 in terms of its evaluations of eating disorder pathologies as measured by EAT-26. There was a significant positive correlation between the EDE-Q-13 scores of the participants and their EAT-26 (including its subscales) scores, and this result was in line with the results of previous studies [39, 40]. The Turkish version of EDE-Q-13 also showed convergent validity. Those with higher eating disorder symptoms appear to have lower body appreciation and life satisfaction. Similarly, Lev-Ari et al. [25] found a negative correlation between total EDE-Q-13 scores and life satisfaction and a positive body image. The BMI values of the participants were positively correlated with their EDE-Q-13 total scores, EDE-Q-13 subscale scores, and EAT-26 scores. Similarly, Prnjak et al. (41) stated that participants with higher BMIs had higher EDE-Q total scores. EAT-26 scores and BMI values also showed a positive, albeit weaker relationship [41]. It may be stated that the relationship between EDE-Q-13 and the scales that were included in this study for comparison is a strong aspect of the scale in the evaluation of eating disorders.

This study had some limitations. First, the assessment of all scales was based on self-report. Second, the body weights and heights of the individuals were also recorded based on their self-reports. Third, the sample was limited to some demographic characteristics. Although the aim was to include equal numbers of male and female participants, the majority of the individuals who agreed to participate in the study consisted of female individuals. Additionally, EDE-Q was validated in clinical and non-clinical samples [21]. Due to its brevity, EDE-Q-13 is a beneficial tool that is appropriate for evaluating clinical samples. In further studies, the validity and reliability of EDE-Q-13 in different populations and clinical evaluations such as anorexia or bulimia nervosa should be examined.

Despite some limitations, the validity and reliability of a short form of EDE-Q were demonstrated for the first time in a Turkish sample with this study. In the present study, it was determined that the Turkish version of EDE-Q-13 possesses high levels of validity and reliability. Both EFA and CFA results showed that it is appropriate to use the Turkish version of EDE-Q-13. EDE-Q-13 takes less time because it is approximately 50% shorter than the original EDE-Q, thereby reducing the burden on both the researcher and the participants. Therefore, it may also prevent the participants from withdrawing due to the time burden. It was also thought that EDE-Q-13 has an advantage because it includes Bingeing and Purging items as a Likert-type scale, which are not available in other short versions, but they were asked in an open-ended form in the original scale [25].

## Conclusion

Scales that are used in the identification of eating disorders, whose prevalence has increased in recent years, are important in preventing the risk of eating disorders. In this study, it was determined that the Turkish version of EDE-Q-13 showed high levels of validity and reliability. The Turkish version of EDE-Q-13 is considered particularly useful for epidemiological studies and large-scale surveys, as it is short, user-friendly, and reliable for assessing eating disorder symptoms, investigating risk factors, and examining the psychological and physical consequences of eating disorders in the Turkish population.

## Data Availability

The datasets used during the present study can be obtained from the corresponding author on reasonable request.

## Abbreviations

EDE-Q: Eating Disorder Examination Questionnaire
EAT-26: Eating Attitudes Test-26
SWLS: Satisfaction with Life Scale
BAS: Body Appreciation Scale
EFA: Explanatory Factor Analysis
CFA: Confirmatory Factor Analysis
ER: Eating Restraint
SWO: Shape and Weight Over-Evaluation
BD: Body Dissatisfaction
BMI: Body Mass Index
WHO: World Health Organization
KMO: Kaiser–Meyer–Olkin
RMSEA: Root Mean Square Error of Approximation
GFI: Goodness-of-Fit Index
AGFI: Adjusted Goodness-of-Fit Index
CFI: Comparative Fit Index
NFI: Normed Fit Index
TLI: Tucker–Lewis Index
SPSS: Statistical Package for the Social Sciences

## References

[1] Martinussen M, Friborg O, Schmierer P, Kaiser S, Øvergård KT, Neunhoeffer AL (2017). The comorbidity of personality disorders in eating disorders: a meta-analysis. Eat Weight Disord.

[2] Qian J, Wu Y, Liu F, Zhu Y, Jin H, Zhang H (2022). An update on the prevalence of eating disorders in the general population: a systematic review and meta-analysis. Eat Weight Disord.

[3] Hay P, Mitchison D (2021). Urbanization and eating disorders: a scoping review of studies from 2019 to 2020. Curr Opin Psychiatry.

[4] Walsh BT (2019). Diagnostic categories for eating disorders: current status and what lies ahead. Psychiatr Clin N Am.

[5] Fairburn CG, Beglin SJ (1994). Assessment of eating disorders: Interview or self-report questionnaire?. Int J Eat Disord.

[6] Mahmoodi M, Moloodi R, Ghaderi A (2016). The Persian version of Eating Disorder Examination Questionnaire and clinical impairment assessment: norms and psychometric properties for undergraduate women. Iran J Psychiatry.

[7] Isomaa R, Lukkarila IL, Ollila T, Nenonen H, Charpentier P, Sinikallio S (2016). Development and preliminary validation of a Finnish version of the Eating Disorder Examination Questionnaire (EDE-Q). Nord J Psychiatry.

[8] Mitsui T, Yoshida T, Komaki G (2017). Psychometric properties of the eating disorder examination-questionnaire in Japanese adolescents. Biopsychosoc Med.

[9] Zohar AH, Lev-Ari L, Bachner-Melman R (2017). The EDE-Q in Hebrew: structural and convergent/divergent validity in a population sample. Isr J Psychiatry.

[10] Rø Ø, Reas DL, Stedal K (2015). Eating disorder examination questionnaire (EDE-Q) in Norwegian adults: discrimination between female controls and eating disorder patients. Eur Eat Disord Rev.

[11] Forsén Mantilla E, Birgegård A, Clinton D (2017). Factor analysis of the adolescent version of the Eating Disorders Examination Questionnaire (EDE-Q): results from Swedish general population and clinical samples. J Eat Disord.

[12] Carrard I, Lien Rebetez MM, Mobbs O, Van der Linden M (2015). Factor structure of a French version of the eating disorder examination-questionnaire among women with and without binge eating disorder symptoms. Eat Weight Disord.

[13] Yucel B, Polat A, Ikiz T, Dusgor BP, Elif Yavuz A, Sertel BO (2011). The Turkish version of the eating disorder examination questionnaire: reliability and validity in adolescents. Eur Eat Disord Rev.

[14] Rand-Giovannetti D, Cicero DC, Mond JM, Latner JD (2020). Psychometric properties of the Eating Disorder Examination-Questionnaire (EDE-Q): a confirmatory factor analysis and assessment of measurement invariance by sex. Assessment.

[15] Jenkins PE, Rienecke RD (2022). Structural validity of the eating disorder examination-questionnaire: a systematic review. Int J Eat Disord.

[16] White HJ, Haycraft E, Goodwin H, Meyer C (2014). Eating disorder examination questionnaire: factor structure for adolescent girls and boys. Int J Eat Disord.

[17] Carey M, Kupeli N, Knight R, Troop NA, Jenkinson PM, Preston C (2019). Eating Disorder Examination Questionnaire (EDE-Q): norms and psychometric properties in UK females and males. Psychol Assess.

[18] Grilo CM, Reas DL, Hopwood CJ, Crosby RD (2015). Factor structure and construct validity of the eating disorder examination-questionnaire in college students: further support for a modified brief version. Int J Eat Disord.

[19] Tobin LN, Lacroix E, von Ranson KM (2019). Evaluating an abbreviated three-factor version of the Eating Disorder Examination Questionnaire in three samples. Eat Behav.

[20] Machado PP, Grilo CM, Rodrigues TF, Vaz AR, Crosby RD (2020). Eating disorder examination–questionnaire short forms: a comparison. Int J Eat Disord.

[21] Berg KC, Peterson CB, Frazier P, Crow SJ (2012). Psychometric evaluation of the eating disorder examination and eating disorder examination-questionnaire: a systematic review of the literature. Int J Eat Disord.

[22] Gideon N, Hawkes N, Mond J, Saunders R, Tchanturia K, Serpell L (2016). Development and psychometric validation of the EDE-QS, a 12 item short form of the Eating Disorder Examination Questionnaire (EDE-Q). PLoS ONE.

[23] Friborg O, Reas DL, Rosenvinge JH, Rø Ø (2013). Core pathology of eating disorders as measured by the Eating Disorder Examination Questionnaire (EDE-Q): the predictive role of a nested general (g) and primary factors. Int J Methods Psychiatr Res.

[24] Kliem S, Mößle T, Zenger M, Strauß B, Brähler E, Hilbert A (2016). The eating disorder examination-questionnaire 8: a brief measure of eating disorder psychopathology (EDE-Q8). Int J Eat Disord.

[25] Lev-Ari L, Bachner-Melman R, Zohar AH (2021). Eating Disorder Examination Questionnaire (EDE-Q-13): expanding on the short form. J Eat Disord.

[26] Tayfur SN, Evrensel A (2020). Investigation of the relationships between eating attitudes, body image and depression among Turkish university students. Riv Psichiatr.

[27] Garner DM, Olmsted MP, Bohr Y, Garfinkel PE (1982). The eating attitudes test: psychometric features and clinical correlates. Psychol Med.

[28] Ergüney-Okumuş FE, Sertel-Berk HÖ (2019). Yeme Tutum Testi kısa formunun (YTT-26) Üniversite örnekleminde Türkçeye uyarlanması ve psikometrik özelliklerinin değerlendirilmesi. Psikoloji Çalışmaları.

[29] Diener E, Emmons RA, Larsen RJ, Griffin S (1985). The satisfaction with life scale. J Pers Assess.

[30] Dağlı A, Baysal N (2016). Yaşam doyumu ölçeğinin Türkçe’ye uyarlanması: geçerlik ve güvenirlik çalışması. Elektronik Sosyal Bilimler Dergisi.

[31] Avalos L, Tylka TL, Wood-Barcalow N (2005). The body appreciation scale: Development and psychometric evaluation. Body Image.

[32] Bakalım O, Taşdelen-Karçkay A (2016). Body Appreciation Scale: Evaluation of the factor structure and psychometric properties among male and female Turkish university students. Mersin Univ J Fac Educ.

[33] Body Mass Index-BMI [cited 2 May 2022]. https://www.euro.who.int/en/health-topics/disease-prevention/nutrition/a-healthy-lifestyle/body-mass-index-bmi.

[34] Beaton DE, Bombardier C, Guillemin F, Ferraz MB (2000). Guidelines for the process of cross-cultural adaptation of self-report measures. Spine.

[35] Nunnally J, Bernstein I (1994). Psychometric theory.

[36] Simon D, Kriston L, Loh A, Spies C, Scheibler F, Wills C (2010). Confirmatory factor analysis and recommendations for improvement of the Autonomy-Preference-Index (API). Health Expect.

[37] Çokluk Ö, Şekercioğlu G, Büyüköztürk Ş. Sosyal bilimler için çok değişkenli istatistik: SPSS ve LISREL uygulamaları. Pegem Atıf İndeksi. 2018;001–414.

[38] Lawshe CH (1975). A quantitative approach to content validity. Pers Psychol.

[39] He J, Sun S, Fan X (2021). Validation of the 12-item Short Form of the Eating Disorder Examination Questionnaire in the Chinese context: confirmatory factor analysis and Rasch analysis. Eat Weight Disord Stud Anorex Bulim Obes.

[40] Asl EM, Mahaki B, Khanjani S, Mohammadian Y (2021). Assessment of eating disorder psychopathology: the psychometric properties of the Persian version of the Eating Disorder Examination Questionnaire Short Form. J Res Med Sci Off J Isfahan Univ Med Sci.

[41] Prnjak K, Jukic I (2021). Development and validation of the Croatian version of the Eating Disorder Examination Questionnaire in a community sample. Eat Weight Disord Stud Anorex Bulim Obes.

